# Soil CO_2_ emission and soil attributes associated with the microbiota of a sugarcane area in southern Brazil

**DOI:** 10.1038/s41598-021-87479-2

**Published:** 2021-04-15

**Authors:** Mara Regina Moitinho, Daniel De Bortoli Teixeira, Elton da Silva Bicalho, Alan Rodrigo Panosso, Antonio Sergio Ferraudo, Gener Tadeu Pereira, Siu Mui Tsai, Beatriz Maria Ferrari Borges, Newton La Scala Jr.

**Affiliations:** 1 Brazilian Biorenewables National Laboratory (LNBR), Brazilian Center for Research in Energy and Materials (CNPEM), Rua Giuseppe Maximo Scolfaro 10000, Campinas, São Paulo 13083-100 Brazil; 2 Center for Agricultural Sciences, University of Marília (UNIMAR), Av. Hygino Muzzy Filho 1001, Marília, São Paulo 17525-902 Brazil; 3 School of Agricultural and Veterinarian Sciences (FCAV), São Paulo State University (Unesp), Via de Acesso Prof. Paulo Donato Castellane s/n, Jaboticabal, São Paulo 14884-900 Brazil; 4 University of São Paulo (USP), Center of Nuclear Energy in Agriculture (CENA), Av. Centenário 303, Piracicaba, São Paulo 13416-000 Brazil

**Keywords:** Climate-change impacts, Climate-change mitigation

## Abstract

The spatial structure of soil CO_2_ emission (FCO_2_) and soil attributes are affected by different factors in a highly complex way. In this context, this study aimed to characterize the spatial variability patterns of FCO_2_ and soil physical, chemical, and microbiological attributes in a sugarcane field area after reform activities. The study was conducted in an Oxisol with the measurement of FCO_2_, soil temperature (Ts), and soil moisture (Ms) in a regular 90 × 90-m grid with 100 sampling points. Soil samples were collected at each sampling point at a depth of 0–0.20 m to determine soil physical (density, macroporosity, and microporosity), particle size (sand, silt, and clay), and chemical attributes (soil organic matter, pH, P, K, Ca, Mg, Al, H + Al, cation exchange capacity, and base saturation). Geostatistical analyses were performed to assess the spatial variability and map soil attributes. Two regions (R1 and R2) with contrasting emission values were identified after mapping FCO_2_. The abundance of bacterial 16S rRNA, *pmo*A, and *nif*H genes, determined by real-time quantitative PCR (qPCR), enzymatic activity (dehydrogenase, urease, cellulase, and amylase), and microbial biomass carbon were determined in R1 and R2. The mean values of FCO_2_ (2.91 µmol m^−2^ s^−1^), Ts (22.6 °C), and Ms (16.9%) over the 28-day period were similar to those observed in studies also conducted under Oxisols in sugarcane areas and conventional soil tillage. The spatial pattern of FCO_2_ was similar to that of macropores, air-filled pore space, silt content, soil organic matter, and soil carbon decay constant. No significant difference was observed between R1 and R2 for the copy number of bacterial 16S rRNA and *nif*H genes, but the results of qPCR for the *pmo*A gene presented differences (p < 0.01) between regions. The region R1, with the highest FCO_2_ (2.9 to 4.2 µmol m^−2^ s^−1^), showed higher enzymatic activity of dehydrogenase (33.02 µg TPF g^−1^ dry soil 24 h^−1^), urease (41.15 µg NH_4_–N g^−1^ dry soil 3 h^−1^), amylase (73.84 µg glucose g^−1^ dry soil 24 h^−1^), and microbial biomass carbon (41.35 µg C g^−1^ soil) than R2, which had the lowest emission (1.9 to 2.7 µmol m^−2^ s^−1^). In addition, the soil C/N ratio was higher in R2 (15.43) than in R1 (12.18). The spatial pattern of FCO_2_ in R1 and R2 may not be directly related to the total amount of the microbial community (bacterial 16S rRNA) in the soil but to the specific function that these microorganisms play regarding soil carbon degradation (*pmo*A).

## Introduction

Carbon loss is one of the main threats to soils, as it is listed by FAO as one of the main aspects associated with soil conservation^1^. A decrease in organic carbon affects soil structure, making it more sensitive to compaction and reducing its capacity to retain water and nutrients^2^.

According to data presented in the IPCC special report on climate change and land use^3^, the increase in soil organic carbon is considered one of the most economically viable options for adapting and mitigating climate change and combating desertification, soil degradation, and food insecurity. The authors argue that maintaining existing carbon stocks and increasing the potential for sequestration through sustainable soil management practices are the best strategy to offset part of global emissions and simultaneously provide various environmental benefits, increasing soil quality and, consequently, the net primary productivity, thus reducing the economic pressure to convert native lands into agricultural production areas.

The intense soil tillage carried out during field reforms in sugarcane (*Saccharum* spp.) areas modifies soil architecture, increasing the roughness of its surface and the amount of air-filled pore space, creating different aggregate size distributions and changing soil moisture and temperature regimes^4,5^.

This management practice leads to complex interactions between water availability, temperature increase, and higher aeration and substrate for microorganisms in the surface soil layers, affecting the mineralization activity, which regulates soil carbon accumulation^6^. In addition to tillage activities, the limestone used for soil pH correction can also significantly increase soil CO_2_ emissions^7–11^.

The spatial variability of soil respiration has been associated with patterns of different soil attributes. The quantity and quality of soil organic matter^12–14^, total porosity and air-filled pore space^15–17^, oxygenation^18^, soil pH^19^, microbial biomass^20^, and soil texture^21,22^ are directly associated with soil CO_2_ emissions and influenced by soil management.

Studies conducted in sugarcane areas have been usually limited to investigating the spatial pattern of soil CO_2_ emission under different crop management systems^16,17,23,24^. However, few of these studies have assessed this pattern in areas after sugarcane field reform. Soils without vegetation cover in which even the sugarcane ratoons are eliminated have exclusive heterotrophic soil respiration^15,25^. Although the spatial pattern of the soil microbiota is often associated with plant species and biomass^26^, transformations that occur in this environment due to the physicochemical soil management interfere significantly with the metabolic and functional capacity of this community and the presence of specific microorganisms^27^.

Although the study of the spatial variation of soil CO_2_ emission on small and medium scales (sampling grid from 1 m^2^ to 1 ha) has been carried out by several authors, it is always associated with soil physicochemical attributes^16,17,23,25,28,29^ or spatial patterns of vegetation cover in case of forests^20,30^. On the other hand, the spatial variability of biological attributes is only studied on small scales, which involve different soil management and use^15,20,31^, mainly due to the high cost of their quantification, preventing the study of a large number of samples. Microbiology is essential to the CO_2_ production process in the soil and is possibly related to its emission, which has been neglected.

In this context, this study hypothesized that the spatial variation of soil CO_2_ emission could be explained by changes in soil microbiological attributes, in addition to the variability of soil physical and chemical attributes. Thus, this study aimed to characterize the spatial variability of soil CO_2_ emission and soil physical, chemical, and microbiological attributes in a sugarcane area under bare soil conditions.

## Material and methods

### Location and characterization of the study area

This study was conducted in an agricultural area destined for sugarcane production located in Barrinha, São Paulo State, Southern Brazil. The geographical coordinates of the area are 21°13′ S and 48°07′ W, with a mean altitude of 555 m above sea level. The soil is classified as an Oxisol (Eutrorthox, USDA Soil Taxonomy), with a slope lower than 0.5%. The area occupies a geomorphological province named basaltic cuestas. The regional climate is defined as B_1_rB′_4_a′ according to the Thornthwaite classification, that is, a humid mesothermal climate with a small water deficit and summer evapotranspiration lower than 70% of the annual evapotranspiration^32^. The mean annual precipitation registered during the last 30 years was 1560 mm, concentrated from October to March, and a mean annual temperature of 22.2 °C.

Sugarcane has been cultivated in the area for more than 10 years under the mechanized harvesting system, with the last field reform carried out in 2008. Ratoons were mechanically eliminated before tillage operations using an implement consisting of rotary hoes, which cuts the soil and ratoons at a high rotation, and throws them against an impact plate of the implement at a high speed, with the breaking of clods and separation of ratoons from the soil. Limestone and gypsum were applied in the area after ratoon elimination, followed by a leveling harrow operation.

Soil tillage operation consisted of the use of an offset disc harrow with 28-notched discs of 28″, half of them in the front section and the other half in the rear section, a working width of 3.5 m, and a working depth of 0.25 m. Two operations were carried out with this implement at a mean speed of approximately 7 km h^−1^, the second operation immediately after the first one to simulate the effect of the disc harrow^11^. A regular grid of 90 × 90 m containing 100 points with minimum distances between sampling points of 10 m was installed after these operations.

### Measurements of soil CO_2_ emission, soil temperature, and soil moisture

Ten measurements were performed over a period of 28 days (September 24, 26, and 30 and October 3, 7, 10, 14, 16, 18, and 21, 2013) from 8:00 to 10:50 h. A portable LI-8100 automated soil CO_2_ flux system (LI-COR, Lincoln, NE, United States) was used to measure the soil CO_2_ emission (FCO_2_) in the experimental area. This system monitors changes in CO_2_ concentration inside a closed chamber using optical absorption spectroscopy in the infrared spectrum. The chamber is a closed system with an 854.2-cm^3^ internal volume and an 83.7-cm^2^ circular soil contact area coupled to PVC collars previously inserted at each sampling points to a depth of 3 cm. In the measurement mode, FCO_2_ was determined inside the chamber every 2.5 s and 1.5 min were required to record it at each point^11,16,17,23,33^.

Soil temperature (Ts) was measured using a temperature sensor from the LI-8100 system, which consists of a 20-cm probe that was inserted into the soil near the PVC collars. Similarly, soil moisture (Ms) was measured using a Time Domain Reflectometry (TDR) system (Hydrosense, Campbell Scientific Inc., Logan, UT, United States), which consists of two 12-cm probes that were also inserted into the soil near the PVC collars. The measurements of Ts and Ms were carried out concomitantly with FCO_2_ assessments.

### Determination of soil physical and chemical attributes

Soil samplings were performed at the end of FCO_2_ assessments at a depth of 0–20 cm and each grid point. Therefore, 100 disturbed soil samples were collected for chemical analysis, and 100 undisturbed soil samples were taken for physical analysis. For soil chemical analysis, samples were collected with a Dutch auger, being then dried, decloded, and sieved through a 2-mm mesh sieve. The analyses included soil organic matter content (SOM), phosphorus availability (P), K, Ca, Mg, and H + Al contents^34^, sum of bases (SB), and cation exchange capacity (CEC). The total organic carbon (TOC) was estimated by dividing SOM by 1.724. The total soil nitrogen (N) was quantified by the Kjeldahl method^35^.

The particle size distribution of sand, silt, and clay were determined by the pipette method using 0.1 mol L^−1^ NaOH as a chemical dispersant and low rotation mechanical stirring for 16 h^36^. Fractions containing particles larger than 0.1 mm were separated by sieving (0.105 mm sieve) and those of smaller size by sedimentation, according to the Stokes law; silt was determined by difference. Soil bulk density (Ds) was determined from undisturbed soil samples collected with a sampler adapted to cylinders with an internal diameter of 5 cm and height of 4 cm. The total pore volume (TPV) was calculated based on Ds, with the pore size distribution determined by a porous plate funnel under a 60-cm water column tension in previously saturated samples. The pore volume retained in the sample corresponded to the micropores, and the difference calculated between TPV and micropores corresponded to the macropores^36^. Air-filled pore space (AFPS) was calculated as the difference between the porosity fraction filled with water (Ms) and TPV.

Soil carbon stock (Cstock) was calculated based on the methodology described by Veldkamp^37^, using Eq. (1).

1 $${\text{Cstock}} = \frac{{{\text{OC}} \times {\text{Ds}} \times {\text{E}}}}{10}$$

where Cstock is the soil carbon stock (Mg ha^−1^), OC is the organic carbon content (g kg^−1^), Ds is the soil bulk density (Mg m^−3^), and E is the soil layer depth (20 cm). Soil carbon stability in the sugarcane field reform area was obtained by Eq. (2).

2 $${\text{k}} = \frac{{{\text{C}}{-}{\text{CO}}_{2} }}{{{\text{Cstock}}}}$$

where *k* is the soil carbon decay constant and C–CO_2_ is the labile carbon emitted into the atmosphere as CO_2_.

### Microbiological analyses

Two regions with distinct emission potentials were identified after FCO_2_ interpolation, being named as R1 (Region 1) and R2 (Region 2), from which soil samples were collected for the microbiological characterization.

#### Quantitative real-time PCR

##### DNA extraction from environmental samples

Soil samples were collected from nine points at each experimental area (R1 and R2). Each of these points came from composite sampling, which means that 15 samples were collected in the area around a central point to obtain its results. Pre-sterilized PVC tubes with dimensions of 50 cm in length and 5 cm in diameter were used for the soil sampling. These PVC tubes were inserted vertically into the soil to collect soil samples. Subsequently, all the PVC tubes were sealed to avoid losses, stored in iceboxes, and sent to the Laboratory of Biochemistry of Microorganisms and Plants of the School of Agricultural and Veterinarian Sciences of the São Paulo State University (FCAV-UNESP), Jaboticabal campus, São Paulo State, Brazil, where they were stored in an ultra-freezer at − 80 °C to be used for genomic DNA extraction.

The genomic DNA of the soil collected at each region was extracted by the FastDNA SPIN Kit for Soil (MP Biomedicals), following the manufacturer’s instructions, and stored at − 20 °C. A 5-µL aliquot was submitted to electrophoresis at 1% (w/v) agarose gel stained in GelRed (Uniscience) in SB buffer to confirm the DNA extraction quality^38^. A 2-µL Low DNA Mass Ladder (Invitrogen) was used as a molecular standard. This gel was submitted to an electric field of 85 V for approximately 30 min. Subsequently, the DNA was quantified in a Nanodrop 2000c spectrophotometer (Thermo Scientific) with an optical density ratio of 1.0 at 260 nm (OD_260_) equal to 50 ng of DNA μ^−1^^39^.

##### Detection and quantification of bacterial 16S rRNA, pmoA, and nifH genes by quantitative real-time PCR

Quantitative real-time PCR reactions were performed on the StepOnePlus Real-Time PCR System (Applied Biosystems) for bacterial 16S rRNA, *pmo*A, and *nif*H genes in the SYBER Green I system. The established conditions for each gene are shown in Table 1. A melting curve was added at the end of each reaction under the following conditions: 95 °C for 15 s and primer annealing temperature for 1 min, gradually increased with the reading of the data at every 0.7 °C until 95 °C.

**Table 1:** Primers used for the amplification of bacterial 16S rRNA, *pmo*A, and *nif*H genes.

Target gene	Primer	Sequence (5′–3′)	Fragment size (bp)	Reference	Amplification conditions
Bacterial 16S rRNA	Eub 338f	ACTCCTACGGGAGGCAGCAG	180	Bakke et al. (2011)^40^	95 °C for 10 min; 40 cycles of 94 °C for 15 s, 56 °C for 30 s, and 72 °C for 45 s
	Eub 518r	ATTACCGCGGCTGCTGG			
*pmoA*	A189 f	GGN GAC TGG GAC TTC TGG	273	Degelmann et al. (2010)^41^	95 °C for 5 min; 40 cycles of 94 °C for 20 s, 55 °C for 1 min, and 72 °C for 45 s
	A682 r	GAA SGC NGA GAA GAA SGC			
*nifH*	*nifH*	AAA GGY GGW ATC GGY AAR TCC ACC AC	457	Wallenstein and Vilgalys (2005)^42^	95 °C for 5 min; 40 cycles of 95 °C for 30 s, 59 °C for 30 s, 72 °C for 1 min, and 72 °C for 1 min
		TTG TTS GCS GCR TAC ATS GCC ATC AT			

Standard curves were obtained by performing amplification with the copy number of template DNA of *Pseudomonas fluorescens* for the bacterial 16S rRNA gene, environmental sample DNA for the *pmo*A gene, and *Bradyrhizobium liaoningense* for the *nif*H gene. The data obtained by amplification of the DNA extracted from soil were interpolated to determine the copy number of the genes under study. The real-time PCR reaction for each gene was prepared at a final volume of 10 µL, containing 5 µL SYBR Green ROX qPCR Master Mix (Thermo Scientific), 5 pmol of each forward and reverse primers, 1 µL DNA of test sample, and sterile ultrapure water. The data of qPCR were obtained by using the StepOne Software 2.2.2 (Applied Biosystems), being subsequently exported to Excel (Microsoft) to calculate the number of gene copies per gram of dry soil.

##### Microbial biomass carbon and soil enzymatic activity

Microbial biomass carbon (MBC) was determined through the irradiation-extraction method, as proposed by Islam and Weil^43^ and adapted by Barbosa^44^. The enzymatic activity of urease and dehydrogenase was determined according to McGarity and Myers^45^ and Casida et al.^46^, respectively. Moreover, the enzymatic activity of amylase was determined according to Barbosa^44^, in which substrate extraction was carried out by the Cole (1977) method, followed by the determination of reducing sugars by the Somogyi^47^ method. The enzymatic activity of cellulase was determined following the method proposed by Kanazawa and Miyashita^48^ and the reducing sugar method of Somogyi^47^.

### Data processing and statistical analysis

The data were initially analyzed using descriptive statistics (mean, standard error of the mean, standard deviation, minimum, maximum, coefficient of variation, skewness, and kurtosis). The analysis of variance was performed using the software R^49^. The spatial dependence was analyzed using geostatistical techniques, with the estimate of the experimental variograms and permissible model adjustments. The variogram was estimated under the assumption of the intrinsic hypothesis by Eq. (3).

3 $$\hat{\gamma }(h) = \frac{1}{2N(h)}\sum\limits_{i = 1}^{N(h)} {\left[ {z(x_{i} + h) - z(x_{i} )} \right]}^{2}$$

where *N*(*h*) is the number of pairs of points of the experimental observations *Z*(*x*_*i*_) and *Z*(*x*_*i*_ + *h*) separated by the *h* distance.

The parameters nugget effect (C_0_), sill (C_0_ + C_1_), and range (a) were estimated for each variable in the adjustment of mathematical models to the experimental variograms. The spatial dependence index (SDI) was used to classify the spatial dependence as weak, moderate, or strong, according to Seidel and Oliveira^50^. This index considers the C_0_, a, and contribution (C_1_) values, the variogram model, and the maximum distance between sampling points. The choice of the best fit to the experimental variogram was based on the lowest sum of squared residuals, the highest coefficient of determination (R^2^), and the cross-validation through the calculation of the root mean square error (RMSE)^51,52^.

The estimate of ordinary kriging (OK) at the non-sampled point $x_{0}$ is given by Eq. (4).

4 $$\hat{z}(x_{0} ) = \sum\limits_{i = 1}^{n} {\lambda_{i} z(x_{i} )}$$

in which $\hat{z}(x_{0} )$ is the OK estimate at the point $x_{0}$, $z(x_{i} )$ is the neighboring value at the site $x_{i} ,\,\,i = 1,\,2,\,...,\,n$, and $\lambda_{i}$ is the weight of observations associated with the neighboring values, estimated based on the adjusted variogram. The construction of maps of spatial patterns (interpolation by OK) was performed using the software Surfer version 9.11.947 (Golden Software Inc., Golden, CO, United States).

Soil CO_2_ emission and the data analyzed in the regions R1 and R2 were submitted to the multivariate exploratory analyses of hierarchical clustering and principal components. The hierarchical clustering analysis is an exploratory multivariate technique to assemble the sample units into groups, allowing the homogeneity within the group and the heterogeneity between groups. The structure of groups in the data is found in a dendrogram constructed with the similarity matrix between samples^53^ using the Euclidean distance, and the linkage of groups was conducted by the Ward method. Hotelling’s T^2^ test was performed to test a possible significant difference between groups observed in the dendrogram. Heatmaps were generated using the Euclidean distance as the distance method, being processed using the package *gplots* in the software R^49^.

Principal component analysis (PCA) is also an exploratory multivariate technique that condenses the information contained in a set of original variables into a smaller set consisting of new latent variables, preserving a relevant amount of original information. The new variables are the eigenvectors (principal components) generated by linear combinations of the original variables, constructed with the eigenvalues of the covariance matrix^54^. Principal components whose eigenvalues were higher than the unity were considered in this analysis, according to the criterion established by Kaiser^55^. The coefficients of linear functions, which define the principal components, were used to interpret their meaning using the signal and relative size of coefficients as an indication of the weight to be assigned to each variable. Only coefficients with high values, usually those higher than or equal to 0.70 in absolute value, were considered in the interpretation. The multivariate analyses were processed using the software Statistica 7.0 (StatSoft Inc., Tulsa, OK, United States).

## Results and discussion

The mean value of FCO_2_ (2.91 µmol m^−2^ s^−1^) over the 28-day period (Table 2) was similar to that observed by Iamaguti et al.^11^ in a study also conducted on an Oxisol in sugarcane areas under the most intense soil tillage (conventional soil tillage). Moreover, the values observed for Ts (22.6 °C) and Ms (16.95%) were in accordance with the values reported in other studies carried out after sugarcane field reform^8,9^. These values are consistent with the characteristics of areas under reform, as soil tillage provides low soil moisture values and high soil temperatures.

**Table 2:** Descriptive statistics of soil physical and chemical attributes assessed in the sugarcane field reform area.

Attribute	Mean	SE	SD	Minimum	Maximum	CV	Skewness	Kurtosis
FCO_2_ (µmol m^−2^ s^−1^)*	2.91	0.06	0.56	1.83	5.28	19.33	0.87	2.17
Ts (°C)*	22.58	0.06	0.59	20.01	23.34	2.62	− 1.81	4.4
Ms (%)*	16.94	0.17	1.74	13.90	21.10	10.29	0.49	− 0.52
Ds (g cm^−3^)	1.33	0.01	0.09	1.08	1.57	6.91	− 0.25	0.55
Macro (%)	11.08	0.48	4.80	1.95	24.62	43.35	0.75	0.17
Micro (%)	39.55	0.48	4.82	26.37	45.82	12.18	− 1.21	0.78
TPV (%)	50.63	0.45	4.53	31.14	68.56	8.94	0.40	8.1
AFPS (%)	33.68	0.51	5.09	12.34	54.46	15.10	0.00	6.38
Clay (%)	61.58	0.48	4.83	50.80	68.80	7.85	− 0.58	− 0.78
Silt (%)	20.23	0.34	3.36	14.96	34.23	16.62	1.29	2.36
Sand (%)	17.20	0.16	1.57	13.40	20.36	9.11	− 0.23	− 0.04
SOM (g dm^−3^)	32.17	0.34	3.40	24.00	43.00	10.56	0.32	0.27
Cstock (Mg ha^−1^)	48.08	0.59	5.85	32.40	63.49	12.99	0.10	− 0.1
*k* (d^−1^)	0.0007	0.00	0.0002	0.0003	0.0012	23.03	0.7020	0.8224
pH (CaCl_2_)	5.01	0.03	0.26	4.40	5.50	5.23	− 0.24	− 0.37
P (mg dm^−3^)	28.13	3.21	32.08	9.00	188.00	114.05	3.06	9.39
K (cmol_c_ dm^−3^)	0.15	0.00	0.05	0.05	0.30	31.18	0.88	0.91
Ca (cmol_c_ dm^−3^)	3.36	0.26	2.64	1.01	25.15	78.54	6.19	48.07
Mg (cmol_c_ dm^−3^)	1.21	0.06	0.57	0.40	3.72	47.07	1.93	5.07
Al (cmol_c_ dm^−3^)	0.19	0.03	0.31	0.00	1.27	162.16	1.93	3.47
H + Al (cmol_c_ dm^−3^)	49.33	1.22	12.23	8.74	78.98	24.78	− 0.36	0.74
SB (cmol_c_ dm^−3^)	4.72	0.31	3.13	1.54	29.00	66.26	5.30	37.4
CEC (cmol_c_ dm^−3^)	8.88	0.29	2.88	6.48	31.77	32.46	5.62	40.84
V (%)	50.67	1.22	12.23	21.02	91.26	24.13	0.36	0.74

N = 100, except for *, in which N = 900. *SE *standard error of the mean, *SD *standard deviation, *CV *coefficient of variation (%), *FCO*_*2*_ soil CO_2_ emission, *Ts *soil temperature, *Ms *soil moisture, *Ds *soil bulk density, *Macro *macroporosity, *Micro *microporosity, *TPV *total pore volume, *AFPS *air-filled pore space, *Clay *clay content, *Silt *silt content, *Sand *sand content, *SOM *soil organic matter, *Cstock *soil carbon stock, *k* soil carbon decay constant, *P *phosphorus content, *K *exchangeable potassium content, *Ca *exchangeable calcium content, *Mg *exchangeable magnesium content, *Al *aluminum content, *H + Al *potential acidity, *SB *sum of bases, *CEC *cation exchange capacity, *V *base saturation.

Tillage operations fractionate soil and incorporate air into it, affecting its temperature regime and accelerating the drying process^8^. Soil tillage also mixes the surface layer, more fertile and rich in organic matter, with deeper soil layers, which is associated with better aeration conditions, adequate moisture, high temperatures, increases in FCO_2_ due to favorable conditions to the aerobic microbial activity in the decomposition of soil organic matter^56^, and consequent release of CO_2_ from soil to the atmosphere^7,57,58^.

In addition, soil aggregate breakdown due to mechanical tillage activities provides high emissions due to the release of the CO_2_ stored in deeper soil layers, an effect usually observed in the short term, which comprises intervals from five^59^ to 24 h after soil tillage^4^. Soil physical attributes and crop growth are also affected by management systems^2^. Soil density and total porosity reflect the impact to the soil caused by tillage systems and the machinery traffic in the area^4^. The soil under study had a high density (1.33 g cm^−3^) and predominance of micropores (39.55%) compared to macropores (11.08%). Agricultural areas under conventional soil tillage tend to have a high macroporosity and low microporosity^2^, justifying the results found in our study. Moreover, soil management does not change soil texture, and soils with a clayey texture (61.58%) (Table 2) present high microporosity^60^.

The sugarcane field reform area had the following mean values for chemical attributes: SOM of 32.17 g dm^−3^, Cstock of 45.08 Mg ha^−1^, pH of 5.01, P of 28.13 mg dm^−3^, K of 0.15 cmol_c_ dm^−3^, Ca of 3.36 cmol_c_ dm^−3^, Mg of 1.21 cmol_c_ dm^−3^, Al of 0.19 cmol_c_ dm^−3^, H + Al of 49.33 cmol_c_ dm^−3^, SB of 4.72 cmol_c_ dm^−3^, CEC of 8.88 cmol_c_ dm^−3^, and base saturation of 50.67% (Table 2). The variation of soil attributes can be classified according to its coefficient of variation (CV) (Table 2). The CV value of 19.33% observed for FCO_2_, although considered moderate (12% < CV < 24%) according to criteria proposed by Warrick and Nielsen^61^, was low compared to the values observed by Silva-Olaya et al.^8^ and Iamaguti et al.^11^, also conducted after sugarcane field reform.

In addition, the CV values found for microporosity (12.18%), AFPS (15.10%), silt content (16.62%), and Cstock (12.99%) are also considered moderate (12% < CV < 24%). The variables Ts (2.62%), Ms (10.29%), Ds (6.91%), TPV (8.94%), clay content (7.85%), sand content (9.11%), SOM (10.56%), and pH (5.23%) presented CV values considered low (CV < 12%). Moreover, the CV values for P (114.05%), K (31.18%), Ca (78.54%), Mg (47.07%), Al (162.16%), H + Al (24.78%), SB (66.26%), CEC (32.46%), and V (24.13%) are considered high (CV > 24%) (Table 2). Most of these attributes listed by their high CV are affected by soil chemical management. The high CV values of some attributes suggest a high heterogeneity around the mean. This heterogeneity may have several causes, such as the accumulation and distribution of soil particles as a function of the relief shape and water flow and the lack of homogeneity during the chemical management with soil corrective application in the total area. Considering that the study area presents a smooth relief with a slope lower than 0.5%, the chemical management may assist in understanding the high variability of some soil chemical attributes.

In general, classical statistical methods use both measures of central (mean) tendency and measures of dispersion (variance) to describe a given soil attribute, considering that soil variability occurs entirely random and assuming that its attributes have a normal frequency distribution^62^. However, several studies have shown that soil attributes have spatial dependence^16,17,23,63^, and geostatistical techniques are required to capture the spatial pattern of these attributes^16^. In this sense, the geostatistical analysis showed that the models adjusted to the variograms were mainly spherical, except for clay and silt contents, which presented an exponential model (Table 3). Exponential models are best adjusted to erratic phenomena on the small scale, while spherical models describe properties with a high spatial continuity or less erratic in a short distance^51^.

**Table 3:** Models and parameters adjusted to the experimental variograms of soil attributes.

Attribute	Model	C_0_	C_0_ + C_1_	SDI	*a* (m)	SSR	R^2^	RMSE
FCO_2_	Spherical	1.56E−01	2.57E−01	Weak	26.74	6.33E−05	0.97	0.49
Ts	Spherical	1.51E−01	2.30E−01	Weak	25.27	9.44E−05	0.89	0.46
Ms*	Spherical	5.45E−03	8.34E−03	Moderate	53.74	8.86E−07	0.80	1.52
Ds	Spherical	4.84E−03	7.52E−03	Weak	32.35	4.25E−07	0.79	0.08
Macro	Spherical	7.28E+00	1.61E+01	Moderate	24.31	2.42E+00	0.74	3.92
Micro	Spherical	1.36E+01	2.13E+01	Moderate	38.02	5.50E−01	0.98	4.40
TPV	Spherical	3.01E+00	7.00E+00	Moderate	36.97	2.27E−01	0.97	2.34
AFPS	Spherical	7.42E+00	1.67E+01	Moderate	45.00	8.80E−01	0.98	4.08
Clay	Exponential	5.02E+00	2.43E+01	Moderate	24.60	5.03E+00	0.87	4.78
Silt	Exponential	1.94E+00	7.55E+00	Moderate	22.32	5.56E−02	0.99	2.70
Sand	Spherical	3.78E−01	2.46E+00	Strong	53.98	9.79E−02	0.95	1.22
SOM	Spherical	6.89E+00	1.05E+01	Weak	30.87	5.47E−01	0.89	3.18
Cstock	Spherical	1.38E+01	2.87E+01	Moderate	26.06	1.75E+00	0.96	5.07
*k*	Spherical	8.50E−09	1.64E−08	Weak	21.19	1.67E−18	0.84	0.00
pH	Spherical	3.11E−02	5.71E−02	Weak	23.20	8.39E−06	0.95	0.23
P*	Spherical	2.16E−02	2.49E−01	Strong	28.20	4.81E−03	0.69	12.86
K	Spherical	8.67E−04	2.02E−03	Moderate	22.32	8.37E−08	0.67	0.05
Ca*	Spherical	8.44E−02	1.44E−01	Moderate	29.77	6.98E−05	0.92	1.36
Mg*	Spherical	8.10E−02	1.61E−01	Moderate	25.51	1.57E−05	0.99	0.48
Al*	Spherical	2.00E−02	5.57E−02	Moderate	24.06	3.39E−05	0.85	0.30
H + Al	Spherical	6.90E+01	1.15E+02	Weak	25.37	2.64E+01	0.90	10.58
SB*	Spherical	7.01E−02	1.37E−01	Weak	23.19	1.29E−04	0.85	1.86
CEC*	Spherical	3.86E−03	2.85E−02	Moderate	23.74	1.21E−06	0.99	1.56
V	Spherical	7.95E+01	1.39E+02	Weak	27.55	1.11E+02	0.83	11.22

N = 100; *Logarithmic transformation; C_0_ = nugget effect; C_0_ + C_1_ = sill; a = range (m); *SDI *spatial dependence index, classified according to Seidel and Oliveira^50^, *SSR *sum of squared residuals, *RMSE *root mean square error, obtained through cross-validation, *FCO*_*2*_ soil CO_2_ emission, *Ts* soil temperature, *Ms* soil moisture, *Ds* soil bulk density, *Macro* macroporosity, *Micro* microporosity, *TPV* total pore volume, *AFPS* air-filled pore space, *Clay* clay content, *Silt* silt content, *Sand* sand content, *SOM* soil organic matter, *Cstock* soil carbon stock, *k* soil carbon decay constant, *P* phosphorus content, *K *exchangeable potassium content, *Ca *exchangeable calcium content, *Mg *exchangeable magnesium content, *Al *aluminum content, *H + Al *potential acidity, *SB *sum of bases, *CEC *cation exchange capacity, *V *base saturation.

Spherical models^24,25,28,63–66^ or the alternation between spherical and exponential models^67–69^ have been used to describe the spatial variability of FCO_2_. The ranges of soil attributes of the models adjusted to the experimental variograms presented small changes, varying from 22.3 to 35 m, except for Ms (53.7 m), microporosity (38 m), TPV (37 m), AFPS (45 m), and sand content (54 m) (Table 3). A high range value for the spatial variability structure indicates a distribution with high spatial continuity.

Changes in the ranges of spatial variability models of FCO_2_ have been observed between seasons^30,68^ and months^70^, after precipitation events^63,69,71^, or even according to the size of the sampling grid^28^. In a similar study conducted in the same area as the present study, Silva et al.^69^ observed that the space–time variation of soil CO_2_ emission, soil temperature, soil moisture, and soil aeration were affected by three periods related to the same precipitation event. Moreover, the authors incorporated a correlation analysis in the spatial component and identified sites where soil moisture and air-filled pore space were the only factors that influenced soil respiration.

**Figure 1:**
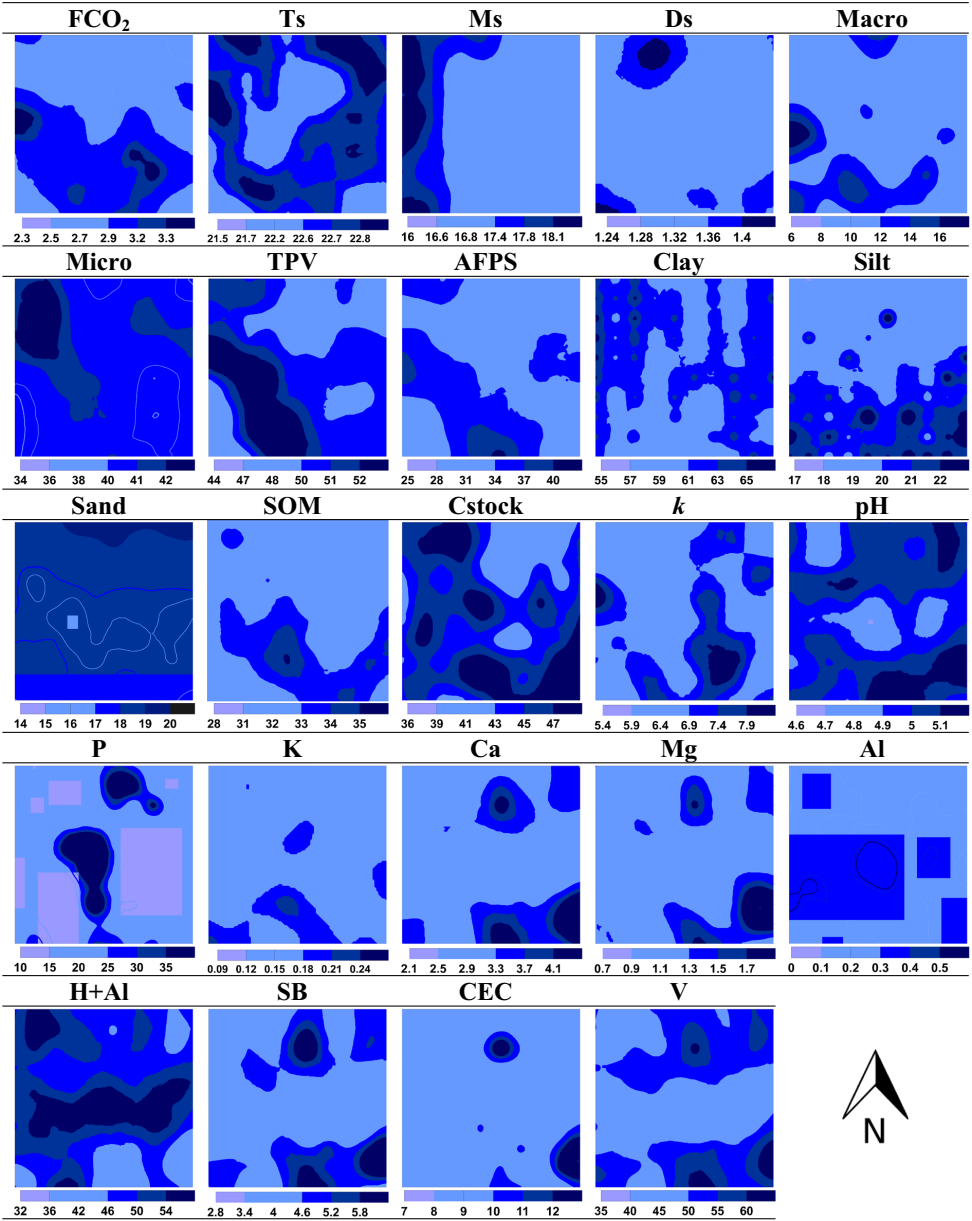
Spatial distribution of soil attributes. FCO_2_ = soil CO_2_ emission (µmol m^−2^ s^−1^); *Ts *soil temperature (°C), *Ms *soil moisture (%), *Ds *soil bulk density (g cm^−3^), *Macro *macroporosity (%), *Micro *microporosity (%), *TPV *total pore volume (%), *AFPS *air-filled pore space (%), *Clay *clay content (%), *Silt *silt content (%), *Sand *sand content (%), *SOM *soil organic matter (g dm^−3^), *Cstock *soil carbon stock (Mg ha^−1^), *k *soil carbon decay constant (d^−1^ × 10^−4^), *P *phosphorus content (mg dm^−3^), *K *exchangeable potassium content (cmol_c_ dm^−3^), *Ca *exchangeable calcium content (cmol_c_ dm^−3^), *Mg *exchangeable magnesium content (cmol_c_ dm^−3^), *Al *aluminum content (cmol_c_ dm^−3^), *H + Al *potential acidity (cmol_c_ dm^−3^), *SB *sum of bases (cmol_c_ dm^−3^), *CEC *cation exchange capacity (cmol_c_ dm^−3^), *V *base saturation (%).

The spatial patterns (maps) of FCO_2_ and other soil attributes (Fig. 1) indicated a subdivision (top and bottom) of the studied area regarding FCO_2_, macroporosity, AFPS, silt content, SOM, K, Ca, Mg, and *k*. This behavior was already expected for *k*, as this constant was determined from FCO_2_ (C–CO_2_). The similarity in the spatial patterns of these attributes indicates a relationship between them. The spatial structure of soil attributes can be affected by numerous factors in a highly complex way due to the spatial and temporal covariation between influencing factors^72^. Also, soil management practices contribute to increasing the variability of soil attributes because their characteristics are affected^33^. Different agricultural practices induce spatial heterogeneity mainly by affecting the ability to retain carbon, water, and nutrients^25,72^. In this context, as observed in the maps, the direct relationship between FCO_2_ and AFPS, macroporosity, SOM, Ca, and Mg is probably due to the activities in the sugarcane field reform, as soil tillage increases the total porosity^4^.

Moreover, soil correction practices increase Ca and Mg contents^56^, promoting favorable conditions for SOM decomposition and, consequently, an increased FCO_2_. Liming carried out before soil tillage operations contributes to increasing FCO_2_ due to the chemical dissolution of carbonate, releasing bicarbonate (2HCO_3_^−^), which turns into CO_2_, besides to changes in biological processes that increase SOM mineralization in response to an increased pH^73,74^. The direct spatial relationship observed between SOM and FCO_2_ occurs because the organic matter represents the main energy reservoir for microorganisms^75,76^. Thus, the increased supply of energy to the microbial metabolism results in an increased release of CO_2_ into the atmosphere. According to^25^, soils without vegetation cover present texture properties that contribute to the spatial and temporal variation of FCO_2_ since they condition the spatial variability of soil water contents. As in our study, these authors also observed a relationship between FCO_2_ and silt content.

Regarding Ms, a division in the east and west was observed in the area. On the other hand, Ts presented a certain homogeneity throughout the area, except for the central region. The maps did not show a relationship between FCO_2_ and Ts and Ms. Several authors have observed that the contribution of these factors is not so great when we analyze the spatial variability of FCO_2_^67,77^. Soil temperature is characterized as one of the main drivers of the temporal variability of soil respiration^18,78^. However, its covariation with soil moisture masks the spatial correlation between soil respiration and soil temperature^25,72,79^.

As observed in the study area, soil respiration is exclusively due to microbial activity without the presence of plants^25^. It increases the variability of the flow of soil greenhouse gases, such as CO_2_, because they are produced or consumed by a great variety of organisms^80^. In this context, most of the studies on FCO_2_ variation have observed relationships with microbial activity^28^, microbial biomass and organic matter content^20^, and variations in the biomass of living and dead organisms associated with the total soil porosity^15^. Although the spatial pattern of soil microorganism diversity is often associated with the vegetation present in the ecosystem^26^, changes occurring in the environment, especially those associated with soil physicochemical management practices, significantly affect its microbiological community^27,81^. This effect occurs because physicochemical management influences the abundance and selection of specific communities of soil microorganisms. These microorganisms are the main agents responsible for GHG production, being highly responsive to any change in the environment. Thus, processes that result in GHG emissions depend directly on the functional diversity and structure of soil microbial communities^82,83^.

**Figure 2:**
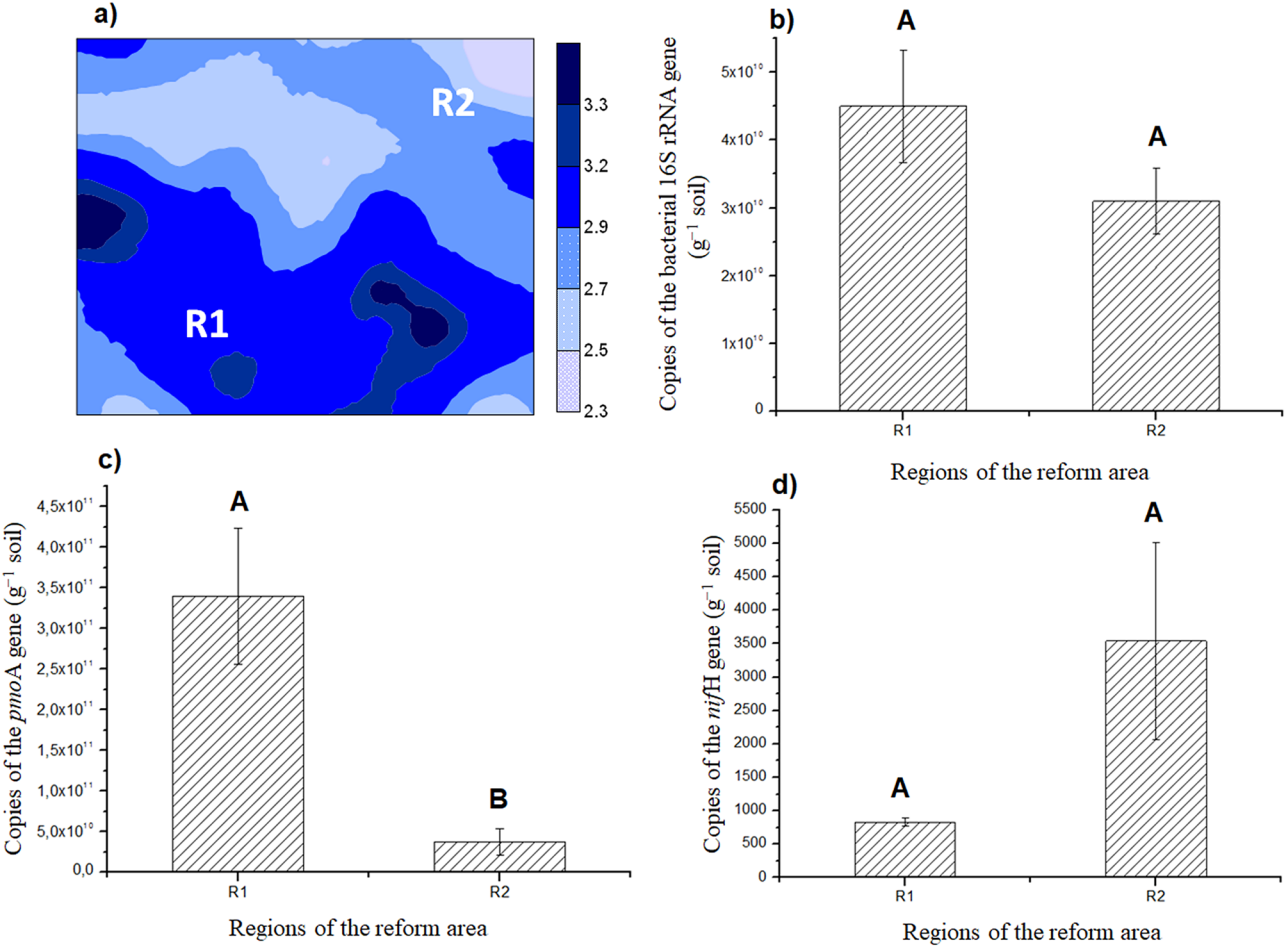
Spatial distribution of FCO_2_ (µmol m^−2^ s^−1^) with identification of two distinct regions in the sugarcane field reform area (R1 and R2) (**a**), graphical representation of both regions with the mean ± standard error of the mean of the copy number of the bacterial 16S rRNA (**b**), *pmo*A (**c**), and *nif*H genes (**d**). Means followed by the same letter do not differ from each other by the Student’s *t* test (p < 0.01).

**Figure 3:**
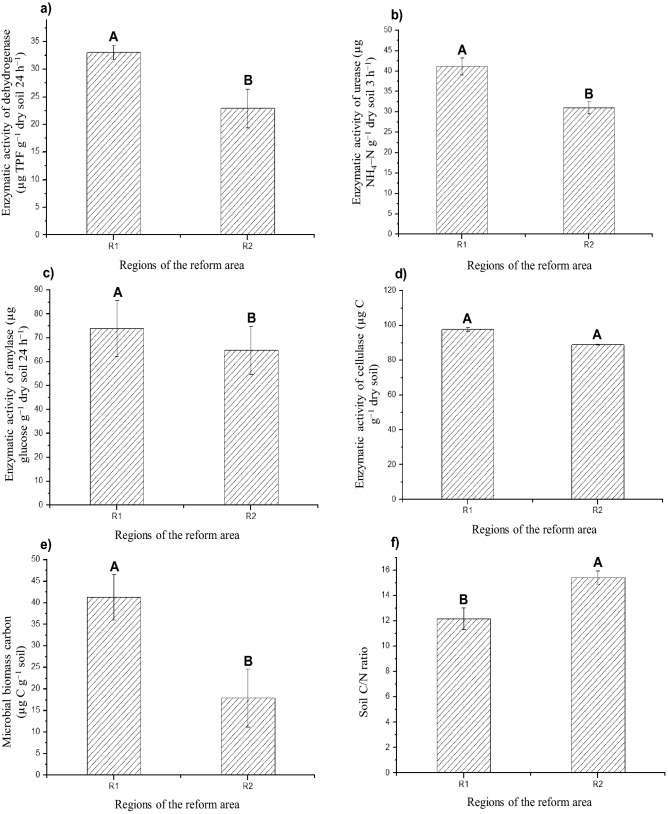
Mean ± standard error of the mean of the enzyme activity of dehydrogenase (**a**), urease (**b**), amylase (**c**), and cellulase (**d**), microbial biomass carbon (**e**), and soil C/N ratio (**f**) in both regions (R1 and R2) of the field reform area. Means followed by the same letter do not differ from each other by the Student’s *t* test (p < 0.01).

The abundance of the bacterial 16S rRNA, *pmo*A, and *nif*H genes (Fig. 2b–d), the enzymatic activity of dehydrogenase, amylase, urease, and cellulase, microbial biomass carbon, and soil C/N ratio were determined after mapping FCO_2_ and identifying the regions R1 and R2 (Fig. 2a), with different potential emissions (Fig. 3). No significant difference was observed in the copy number of the bacterial 16S rRNA gene when the regions R1 (4.3 × 10^9^ g^−1^ soil) and R2 (3.1 × 10^9^ g^−1^ soil) were compared (Fig. 2b). Therefore, the abundance of microorganisms present in the soil of the respective regions was similar. The same was verified regarding the copy number of the *nif*H gene associated with the nitrogen cycle in R1 (823.33 g^−1^ soil) and R2 (3,541.67 g^−1^ soil) (Fig. 2d).

However, a difference (p < 0.01) was observed between R1 (9.5 × 10^4^ g^−1^ soil) and R2 (2.9 × 10^4^ g^−1^ soil) when assessing the abundance of the *pmo*A gene, related to the carbon cycle (Fig. 2c). It indicates that the spatial pattern of FCO_2_ in R1 and R2 may not be directly related to the total amount of the microbial community (16S rRNA) present in the soil, but to the specific function that microorganisms play related to soil carbon degradation (*pmo*A). Also, the abundance of *pmo*A genes increases during the dry season^41^, the period when the present study was conducted. Sengupta and Dick^84^ assessed the methanotrophic bacterial diversity in two different soils under varying land-use practices as determined by high-throughput sequencing of the *pmo*A gene and observed that the study of the diversity of specific groups, such as *pmo*A, can lead us to more accurate interpretations of the correlations between the microbial community and edaphic variables, mainly in studies involving changes in land use aiming at GHG mitigation strategies.

Microorganism populations play an essential role in recycling soil chemical elements by controlling the dynamics of the decomposition and stabilization of carbon^85^ and, consequently, spatiotemporal variability patterns of FCO_2_ into the atmosphere. Moreover, the functional diversity and structure of soil microbial communities are of particular importance for soil functioning as an ecosystem^82,83^. In this sense, the *pmo*A gene has been widely used to detect the presence of methanotrophic bacteria in the soil because they have the ability to use methane as the sole source of carbon and energy and hence play an important role in the global carbon cycle, being potential biodegrading agents^86^.

The determination of soil enzymes also indicates the functional diversity of the microbial community^87^ because soil enzymes are mostly produced from microorganisms. The enzymatic activity has a great potential to indicate biological transformations of the soil in response to changes in its management, as they are sensitive to soil management and directly related to nutrient transformations^88^. Significant differences (p < 0.01) were observed for the enzymatic activity of dehydrogenase (Fig. 3a), urease (Fig. 3b), and amylase (Fig. 3c) when R1 and R2 were compared. On the other hand, no significant difference was observed for the enzymatic activity of cellulase (Fig. 3d). The enzymatic activity of dehydrogenase was higher in R1 (33.02 µg TPF g^−1^ dry soil 24 h^−1^) when compared to R2 (22.90 µg TPF g^−1^ dry soil 24 h^−1^) (Fig. 3a). R1 also presented the highest FCO_2_ (Fig. 2a). Dehydrogenase occurs intracellularly in all living cells of microorganisms, not accumulating extracellularly in the soil, that is, it is only present in living and active organisms^89^.

Determining the enzymatic activity of urease is also important for understanding the spatial pattern of FCO_2_ since this enzyme is responsible for catalyzing the hydrolysis of urea exoenzymes to form CO_2_ and ammonium^90^. The differences (p < 0.01) between R1 (41.15 µg NH_4_–N g^−1^ dry soil 3 h^−1^) and R2 (31.07 µg NH_4_–N g^−1^ dry soil 3 h^−1^) (Fig. 3b), with a higher biosynthesis of urease in R1, reinforces the discussion on the FCO_2_ variability as a function of the higher and lower emission potential be related to soil microbial characteristics in these respective areas.

The enzymatic activity of amylase was also higher in R1 (73.84 µg glucose g^−1^ dry soil 24 h^−1^) than in R2 (64.81 µg glucose g^−1^ dry soil 24 h^−1^) (Fig. 3c). Amylases, among other enzymes synthesized mostly by soil microorganisms, are responsible for SOM mineralization^91^, that is, in this process of organic matter transformation, for instance, the carbon of carbohydrate molecules is released as CO_2_^75^, explaining its higher activity in R1, which presented a higher FCO_2_.

Cellulase is an enzyme responsible for catalyzing cellulose hydrolysis, being partially responsible for the litter decomposition rate and process^92^. Its increase in the soil indicates the entry of the cellulose-enriched substrate into the agrosystem^93^. Cellulase did not differ between the regions R1 and R2 (Fig. 3d), which may be an indication that the sugarcane residue (straw and dead roots) was homogeneously incorporated into the soil at the time of tillage (Fig. 1b), not favoring a given region compared to other regarding the incorporation of residues in the soil.

Soil microbial biomass carbon represents the active and biodegradable fraction of SOM, being partially composed of several microorganism species, such as fungi, bacteria, protozoa, nematodes, and algae, acting as agents in the organic matter mineralization^94^. A significant difference (p < 0.01) was observed for MBC between R1 (41.35 µg C g^−1^ soil) and R2 (17.87 µg C g^−1^ soil) (Fig. 3e). Therefore, R1 presented the highest potential for CO_2_ emission (Fig. 2a) and also the largest active fraction of SOM, being 131% higher in R1 than in R2. On the other hand, the soil C/N ratio was lower in R1 (12.18) than in R2 (15.43) (Fig. 3f). The dynamics of microbial activity is regulated, among other factors, by the C/N ratio since the relationship between these two elements in the soil interferes with the degree of humification and stability of SOM^95^. The fact that this ratio is lower in R1 indicates that the organic matter decomposition process is more accelerated in this region.

**Figure 4:**
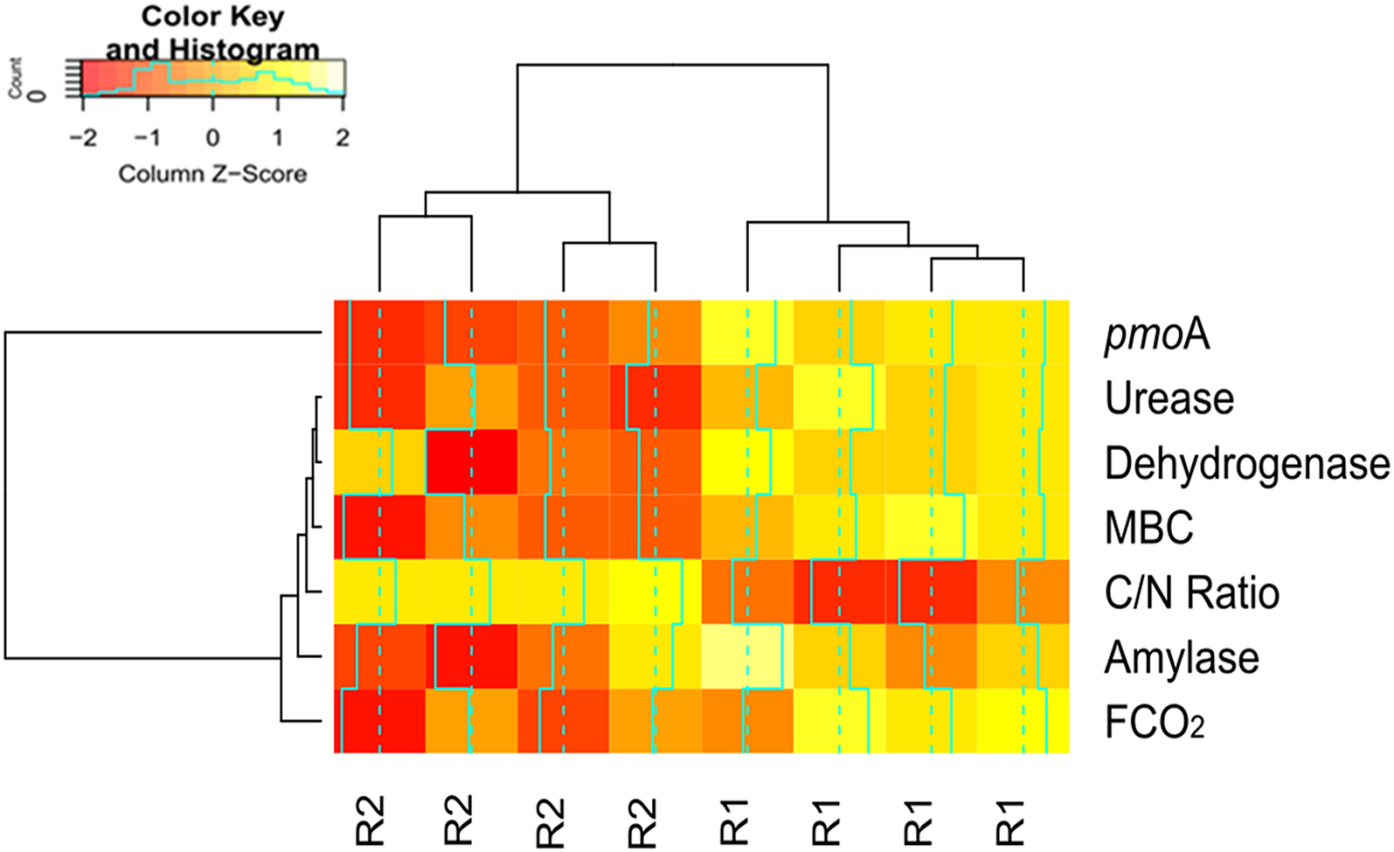
Heatmap graph (r = 0.89; p < 0.0001). Dendrogram showing the hierarchy of groups (regions of the reform area) resulting from the clustering analysis by the hierarchical method. Heatmap shows the variations of soil attributes as a function of management. The dendrogram above the heatmap represents the clustering of regions based on similar patterns of variation. Heatmaps were processed using the package *gplots* in the software R version 3.6.3^49^ (https://cran.r-project.org/bin/windows/base/old/3.6.3/).

**Figure 5:**
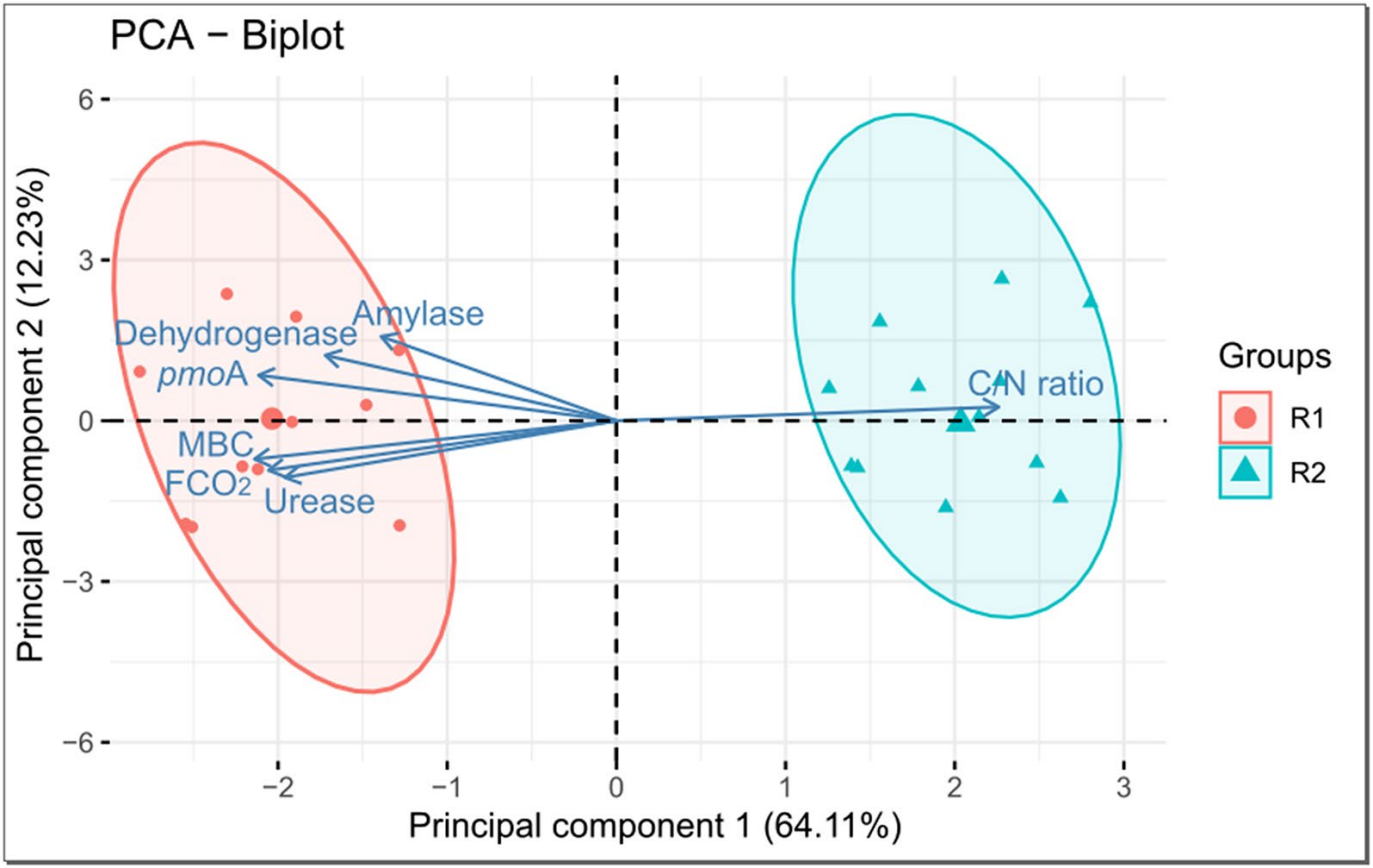
Biplot showing the assessed soil attributes and regions. Amylase = enzymatic activity of amylase; Dehydrogenase = enzymatic activity of dehydrogenase; *pmo*A = *pmo*A functional gene; MBC = microbial biomass carbon; Urease = enzymatic activity of urease; FCO_2_ = soil CO_2_ emission. Principal components were processed using the package *ggplot2* and *factoextra* in the software R version 3.6.3^49^ (https://cran.r-project.org/bin/windows/base/old/3.6.3/).

The analysis of variance indicated differences between the regions R1 and R2 when soil microbiological attributes were univariately assessed (Figs. 2 and 3). In addition, the fact that FCO_2_ was also higher in R1 and lower in R2 (Fig. 2a) allowed establishing indirect relationships. However, the characterization of a pattern or behavior depends on several interactions among the assessed factors. In this context, multivariate analyses of the data can be very efficient since they allow visualizing natural correlations and multiple influences on behavior, especially when using multivariate techniques of interdependence, that is, when no variable or group is treated as dependent or independent^50^. Therefore, when the multivariate analysis was carried out by the hierarchical clustering method, the dendrogram constructed from samples of FCO_2_ and microbiological attributes reaffirmed the existence of two groups: Group 1 (R2), linked to a shorter Euclidean distance, and Group 2 (R1), linked to a larger Euclidean distance (Fig. 4). In addition, a significant effect (F = 150.76; p = 0.007) was observed when carrying out Hotelling’s T^2^ test, confirming that R1 and R2 are distinct groups. Multivariate analysis techniques are only efficient when there is a correlation structure between variables^50^. In our study, this structure is shown in Fig. 5 and Table 4.

**Table 4:** Correlation between soil attributes and the first two principal components (PC1 and PC2).

Principal component	PC1	PC2
Explained variance (%)	64.11*	12.23*
**Correlation**		
Enzymatic activity of dehydrogenase	**− 0.73**	0.43
Enzymatic activity of urease	**− 0.85**	− 0.32
Enzymatic activity of amylase	**− 0.70**	0.51
Microbial biomass carbon (MBC)	**− 0.75**	− 0.16
Soil CO_2_ emission (FCO_2_)	**− 0.76**	− 0.46
*pmo*A gene	**− 0.88**	0.29
Soil C/N ratio	**0.86**	0.03
Interpretation of PC1	FCO_2_ potential as a function of soil microbial diversity	

*Value referring to the percentage of variation of the original set of data retained by the respective principal components. Correlations in bold (higher than 0.70 in absolute value) were considered in the interpretation of the principal component.

Figure 5 shows the biplot with the first two principal components (PC1 and PC2), while the variance assigned to them is shown in Table 4. PC1 represents 64.11% and PC2 represents 12.23% of the total variance of the original data (Fig. 5). All the assessed attributes were retained in PC1 according to the cut-off criterion (> 0.70 in absolute value) considered for interpreting the principal component (Table 4). In PC1, there is a direct dependence relationship among the enzymatic activity of dehydrogenase (0.73), urease (0.85), and amylase (0.70), microbial biomass carbon (0.75), FCO_2_ (0.76), and *pmo*A functional gene (0.88), all directed to the same direction as the samples from R1 (right side of the two-dimensional plane). On the other hand, there is a relationship of indirect dependence of these attributes with the C/N ratio (− 0.86), located in the opposite direction, where the samples of R2 are also located (left side of the two-dimensional plane) (Fig. 5 and Table 4).

These relationships indicate that the process occurring in PC1 may be related to the soil CO_2_ emission potential in R1 and R2. Therefore, for interpretation purposes, PC1 can be termed as FCO_2_ potential as a function of soil microbial diversity because there is a close relationship between the spatial pattern of FCO_2_ and soil microbiota dynamics. This dynamic is regulated by the C/N ratio, that is, the readily available C content, which, as soil pH, has been described in the literature as controlling the taxonomic and functional structure of the microbiota^96–98^. In addition, adequate conditions of aeration, nutrient supply, soil moisture, and soil temperature (Table 2) favored the diversity of soil microorganisms (Fig. 2) and organic matter biodegradation by stimulating the synthesis of soil enzymes (Fig. 3). In this context, all the activities carried out in the reform area, which included soil correction and conventional tillage with residue incorporation, certainly affected the diversity of soil microorganisms and, consequently, FCO_2_ values, providing conditions for the establishment of the regions R1 and R2.

Thus, region R1 presents a higher FCO_2_ potential than R2 (Fig. 2a), which may be associated with a higher soil microbial activity in R1 (Figs. 2c and 3a–e) favored by the lower C/N ratio (Fig. 3f) and better nutritional status provided by the higher content of SOM, Ca, and Mg (Fig. 1), as well as better soil aeration conditions due to the higher percentage of macropores and air-filled pore space in R1 (Fig. 1).

## Conclusions

In sugarcane field reform areas, the spatial patterns of soil CO_2_ emission are similar to those of the attributes macropores, air-filled pore space, silt content, organic matter, and soil carbon decay constant. In our study, differences in soil CO_2_ emission were associated with the dynamics of the microbial activity regulated by the abundance of the *pmo*A gene (related to the carbon cycle) and the enzymatic activity of dehydrogenase, urease, and amylase, microbial biomass carbon, and soil C/N ratio.

Considering that one of the greatest challenges for new directions in world agriculture is to increase the efficiency of conventional practices of soil management, this study reinforces the importance of understanding the spatial pattern of soil attributes and how they influence soil CO_2_ emission dynamics in agricultural areas. This aims for future mitigation actions that involve less intense tillage activities and restrained and homogeneous applications of soil inputs, reducing production costs and the contribution of these activities to the additional CO_2_ emission during the sugarcane field reform period.
